# C-peptide level as predictor of type 2 diabetes remission and body composition changes in non-diabetic and diabetic patients after Roux-en-Y gastric bypass

**DOI:** 10.6061/clinics/2021/e2906

**Published:** 2021-07-26

**Authors:** Roberto de Cleva, Flavio Kawamoto, Georgia Borges, Priscila Caproni, Alex Jones Flores Cassenote, Marco Aurelio Santo

**Affiliations:** Departamento de Gastroenterologia, Hospital das Clinicas HCFMUSP, Faculdade de Medicina, Universidade de Sao Paulo, Sao Paulo, SP, BR.

**Keywords:** Type 2 Diabetes Mellitus, Bariatric Surgery, Diabetes Remission, C-peptide

## Abstract

**OBJECTIVES:**

Several predictors of type 2 diabetes mellitus (T2DM) remission after metabolic surgery have been proposed and used to develop predictive scores. These scores may not be reproducible in diverse geographic regions with different baseline characteristics. This study aimed to identify predictive factors associated with T2DM remission after Roux-en-Y gastric bypass (RYGB) in patients with severe obesity. We hypothesized that the body composition alterations induced by bariatric surgery could also contribute to diabetes remission.

**METHODS:**

We retrospectively evaluated 100 patients with severe obesity and T2DM who underwent RYGB between 2014 and 2016 for preoperative factors (age, diabetes duration, insulin use, HbA1c, C-peptide plasma level, and basal insulinemia) to identify predictors of T2DM remission (glycemia<126 mg/dL and/or HbA1c<6.5%) at 3 years postoperatively. The potential preoperative predictors were prospectively applied to 20 other patients with obesity and T2DM who underwent RYGB for validation. In addition, 81 patients with severe obesity (33 with T2DM) underwent body composition evaluations by bioelectrical impedance analysis (InBody 770®) 1 year after RYGB for comparison of body composition changes between patients with and those without T2DM.

**RESULTS:**

The retrospective analysis identified only a C-peptide level >3 ng/dL as a positive predictor of 3-year postoperative diabetes remission, which was validated in the prospective phase. There was a significant difference in the postoperative body composition changes between non-diabetic and diabetic patients only in trunk mass.

**CONCLUSION:**

Preoperative C-peptide levels can be useful for predicting T2DM remission after RYGB. Trunk mass is the most important difference in postoperative body composition changes between non-diabetic and diabetic patients.

## INTRODUCTION

Type 2 diabetes mellitus (T2DM) is a disease affecting >400 million people worldwide and is among the most important obesity-related diseases (1). Diabetes improvement or remission is considered one of the most important outcomes of the surgical treatment of patients with severe obesity (2). Despite new pharmaceutical treatment options for T2DM, their cost limits their widespread use, especially in underdeveloped countries. Moreover, T2DM control has been inadequate worldwide (3-5).

Severe obesity is characterized by excessive body fat, increased total body water (TBW), and a decreased lean mass. Visceral fat may determine insulin resistance, a characteristic of metabolic syndrome. An individual’s fat-free mass (FFM) plays a key role in promoting insulin sensitivity after Roux-en-Y gastric bypass (RYGB) surgery (6).

Bariatric surgery is currently considered the best treatment for T2DM in patients with severe obesity due to its effects of sustained weight loss and mortality reduction in this population (7-9). Better glycemic control after bariatric surgery is at least partly due to caloric restriction and earlier food passage through the gastrointestinal tract with the release of gut hormones, especially glucagon-like peptide-1 (GLP-1) (10,11).

There are several predictors of T2DM remission after bariatric surgery, such as age, diabetes duration, insulin use, glycated hemoglobin (HbA1c), and C-peptide plasma level. Some of these factors have been used to develop scores to predict T2DM remission after bariatric surgery. The DiaRem score, based on a retrospective review, featured a complete or partial diabetes remission rate of 63% (12). Insulin use before surgery was the strongest negative predictor, but age and HbA1c were also important (12,13). For the ABCD score, age, body mass index (BMI), C-peptide level, and diabetes duration were used to construct a scaling system in which higher scores predict diabetes remission after surgery (14,15). The individualized metabolic surgery (IMS) scoring system was based on independent predictors (number of diabetes medications, insulin use, duration of diabetes, and glycemic control) to guide procedure selection based on long-term (>5 years) diabetes control (16). The authors recommended RYGB for patients with mild or moderate obesity and sleeve gastrectomy for severe obesity according to the efficiency of T2DM remission and risk of each surgical procedure (16). These scores, although validated in prospective cohorts, may not be reproducible in patients from diverse geographic regions with different baseline characteristics since they were developed in the United States (DiaRem and IMS) or Asia (ABCD).

The objective of our study was to identify predictive factors associated with T2DM remission after RYGB in patients with severe obesity. We also hypothesized that the body composition alterations (specifically central fat and FFM) induced by bariatric surgery could differ between non-diabetic and diabetic patients.

## METHODS

The study was divided into retrospective and prospective phases.

### Retrospective phase

In the retrospective phase, 100 patients with severe obesity and T2DM but without renal dysfunction who underwent RYGB between 2014 and 2016 were evaluated using the following preoperative predictors of T2DM remission: age, diabetes duration, insulin use, HbA1c (< or >7.5%), C-peptide plasma level, and basal insulinemia. Diabetes remission was defined as a fasting glycemia<126 md/dL and/or an HbA1c<6.5% in the absence of injectable (insulin or GLP-1 analogs) or oral medications (except metformin) for at least 3 years after RYGB.

### Prospective phase

Three potential preoperative predictors of T2DM remission factors were then applied prospectively to 20 patients with T2DM and severe obesity but without renal dysfunction evaluated before (T0) and 3 years (T2) after RYGB.

### Anthropometric data and body composition

In addition, 81 patients with severe obesity (33 of who also had T2DM) who underwent RYGB between 2016 and 2017 were evaluated for the comparison of postoperative body composition changes between non-diabetic and diabetic patients 1 year after RYGB. Patients who used oral or injectable steroids (n=0) or had hepatitis B (n=0), hepatitis C (n=0), or human immunodeficiency virus infection (n=0) were excluded. Body composition was evaluated using anthropometry and bioelectrical impedance analysis (BIA). Height and body weight were measured with the participants wearing light clothing and no footwear to the nearest 0.5 cm and 0.1 kg, respectively. BMI was calculated by dividing the body weight in kilograms by the height in square meters (kg/m^2^). Body composition was determined by BIA (In Body 770®, Biospace, Cerritos, CA, USA) under constant conditions (proper hydration and same time of day). Each participant was positioned in an orthostatic position on a platform with lower electrodes for the feet and the hands holding onto upper electrodes. BIA determined absolute and relative loss values: BMI, TBW, fat mass, FFM, skeletal muscle mass, and trunk mass (TM).

The current study was performed in the Metabolic and Bariatric Unit, Hospital das Clinicas, University of Sao Paulo Medical School, approved by the local ethical committee, and performed according to the recommendations of the Declaration of Helsinki. Informed consent was obtained from all participants.

### Statistical analysis

All statistical analyses were performed using SPSS version 12.01 (SPSS, Chicago, IL, USA). The results are presented as mean±standard deviation. Statistical significance was set at *p*<0.05. The paired t-test, Mann-Whitney U-test, and Fisher’s exact test were used to determine intergroup differences in numerical data.

## RESULTS

### Retrospective phase

The results of the retrospective phases are presented in Table 1. There was an improvement in the glycemic profile (glycemia<126 md/dL and/or HbA1c<6.5%) with diabetes remission over 3 years postoperatively in 72% of the patients, demonstrating the sustained effect of RYGB on diabetes improvement. The analysis of preoperative factors (age, diabetes duration, insulin use, HbA1c level, C-peptide level, and basal insulinemia) demonstrated that only a C-peptide level>3 ng/dL was a positive predictor (*p*=0.004) of late postoperative diabetes remission.

### Prospective phase

The results of the prospective phase are presented in Table 2. Three potential predictive factors (HbA1c, C-peptide level, and basal insulin) were evaluated prospectively, and C-peptide was confirmed as the only positive predictor of T2DM remission at 3 years after RYGB. Among the six patients without remission, 100% had C-peptide levels <3 ng/mL, while 9 (75%) of 12 patients with diabetes remission had C-peptide levels >3 ng/mL in the preoperative period (*p*=0.014). These data show that a C-peptide level >3 ng/mL is a positive predictor of diabetes remission.

### Anthropometric data and body composition

The body composition results at 1 year after RYGB for non-diabetic and diabetic patients are shown in Table 3. There was a significant difference in body composition only in TM (*p*<0.001), both in absolute and relative loss values.

## DISCUSSION

Our study findings suggest that C-peptide is the only positive predictor of partial or total diabetes remission at least 3 years after RYGB, in agreement with previous studies (17-19). The C-peptide level is a measurement of insulin production and indirectly reflects islet cell mass. Age and diabetes duration are also indirectly related to islet cell involvement in T2DM. C-peptide level may be a powerful predictor of diabetes remission since it reflects pancreatic reserve independent of age or diabetes duration.

The predictive scores developed to predict diabetes remission include age, BMI, diabetes duration, C-peptide level, glycemia, and HbA1c, all of which present some limitations in different geographic areas and ethnically diverse populations. In fact, Tharakan et al. (20) examined an ethnically diverse cohort in the UK and showed that the DiaRem score is useful for predicting diabetes remission only in patients with a low score (high chance of remission). They recommended caution in using this model in areas other than the US Caucasian population.

Moreover, Park Yi (21) reviewed the factors involved in T2DM remission after metabolic surgery and suggested that the three models (DiaRem, ABCD, and IMS scores) need further validation in various ethnic groups to ensure their universal applicability. Thus, these scores may not be adequate for our population.

Most patients who underwent RYGB were young (40-50 years of age). Therefore, age may not be an important factor for predicting diabetes remission in our population.

Determining the duration of diabetes remains a challenge for investigators. Although we considered an important factor in predicting diabetes remission, the readability of diabetes duration data obtained from a patient’s history may be imprecise. Some authors consider that self-reported T2DM duration is consistent with actual T2DM duration (22-24); however, this may not be true in some countries.

Diabetes control is another important predictor of diabetes remission after metabolic surgery (19,25,26). Nevertheless, many patients in Brazil do not have access to new medications because of their high cost for public services. Moreover, many patients were diagnosed with diabetes only months before undergoing bariatric surgery. Therefore, diabetes control was not adequate in our population and cannot be considered a predictor of surgical success for diabetes remission.

In this study, we identified C-peptide as a positive predictive factor that can be useful in guiding postoperative expectations concerning T2DM improvement during long-term follow-up. However, C-peptide is not considered a predictor of T2DM remission in terms of DiaRem and IMS scores. In the ABCD score, only a level >3.9 can predict a positive outcome. A fasting C-peptide level >2.9 ng/mL was considered a predictor for T2DM resolution at 1 year by Dixon et al. (27). In another study, 90% of T2DM patients with a preoperative fasting C-peptide level >3.0 ng/mL had a postoperative HbA1c <6.5% and 74% had complete diabetes resolution after 3.6±0.16 years of follow-up (28).

Clinical and laboratory parameters are closely related, and a single parameter, such as C-peptide, may represent most of the predictive factors. Patients with a long diabetes duration were older and more likely to use insulin. Both conditions were associated with poor pancreatic reserves. C-peptide levels adequately express these three predictors.

Our results confirm that C-peptide is a good predictor of T2DM remission during long-term follow-up.

We also hypothesized that body composition alterations induced by bariatric surgery could also differ between non-diabetic and diabetic patients, especially FFM or TM (29). Nevertheless, non-diabetic and diabetic patients presented the same alterations in body composition after surgery, with differences only in TM. The increase in insulin sensitivity observed after bariatric surgery could be mediated by factors other than FFM, such as muscle extracellular matrix remodeling (6) or visceral fat indicated by TM.

The rates of diabetes remission after bariatric surgery tend to decrease over time, as demonstrated in the SOS and STAMPEDE trials (2,30). Recently, Belligoli et al. (31) suggested that weight regain and diabetes recurrence may be influenced by postoperative eating behaviors and lifestyle modifications to a greater extent than by preoperative predictors of long-term outcomes. Although we agree with these important observations, we consider it important to identify predictors of surgical success and diabetes remission to identify patients at risk of weight regain and diabetes recurrence and institute other treatments (32). On the other hand, understanding changes in body composition can be useful for identifying other predictors in non-diabetic and diabetic patients.

Limitations: The main limitations of our study were its small number of participants and relatively short follow-up. Diabetes remission was defined as glycemia below the diabetes range in the absence of injectable medication therapy or oral medications (except metformin).

## CONCLUSIONS

Preoperative C-peptide level predicts type 2 diabetes remission after RYGB. TM is the most important difference in postoperative body composition changes between non-diabetic and diabetic patients.

## AUTHOR CONTRIBUTIONS

Santo MA and De Cleva R were responsible for the study conception and design, as well as, manuscript drafting and critical revision. Borges G and Caproni P were responsible for body composition evaluation. Kawamoto F, Caproni P and Cassenote AJF were responsible for the data review and glycemic profiles in all patients. All authors participated in the data analysis and interpretation. All authors contributed to and approved the final version of the manuscript. All authors take public responsibility for the manuscript’s content.

## Figures and Tables

**Table 1:** Preoperative predictors of diabetes remission at 3 years after Roux-en-Y gastric bypass in patients with severe obesity and type 2 diabetes mellitus **(retrospective phase).** Diabetes remission was defined as a fasting glycemia<126 md/dL and/or a glycated hemoglobin (HbA1c) level<6.5%.

	DIABETES REMISSION			
Preoperative predictors N=100	YES (n=72)	NO (n=28)	Total	*p*-value
Age				
<60 years	60	22	82	0.385
>60 years	12	6	18	
Diabetes duration^1^				
<10 years	12	8	20	0.537
>10 years	17	10	27	
Insulin use				
Yes	24	13	37	0.162
No	48	15	63	
HbA1c (%)				
<7.5	21	4	25	0.096
>7.5	51	24	75	
C-peptide (ng/mL)^2^				
<3	7	9	16	**0.004**
>3	49	11	60	
Basal insulin (mU/L)^3^				
<25	37	11	48	0.200
>25	19	10	29	

*p*-values were calculated using Fisher’s exact test.

1 Data were unavailable for 53 patients.

2 Data were unavailable for 24 patients.

3 Data were unavailable for 23 patients.

**Table 2:** Preoperative predictors of diabetes remission at 3 years after Roux-en-Y gastric bypass in patients with severe obesity and type 2 diabetes mellitus **(prospective phase).** Diabetes remission was defined as a fasting glycemia level<126 md/dL and/or glycated hemoglobin (HbA1c) level<6.5%.

	DIABETES REMISSION					
Preoperative predictors (N=20)	YES (n=14)	%	NO (n=6)	%	Total	*p*-value*
HbA1c (%)						
<7.5	6	42.8	1	16.7	7	0.354
>7.5	8	57.2	5	83.3	13	
C-peptide (ng/mL)						
<3	5	35.7	6	100	11	**0.014**
>3	9	64.3	0	0	9	
Basal insulin (mU/L)						
<25	9	75.0	4	66.7	13	0.990
>25	3*	25.0	2	33.3	5	

*p*-values were calculated using Fisher’s exact test.

* Data were unavailable for two patients.

**Table 3:** Results of body composition evaluated by bioimpedance analysis 1 year after Roux-en-Y gastric bypass comparing non-diabetic and diabetic (T2DM) patients with severe obesity.

Postoperative (N=81)	NON-DIABETIC (n=48)		DIABETIC (n=33)		*p*-value
	Means±SD	Median (IQR)	Mean±SD	Median (IQR)	
ABSOLUTE VALUES				
BMI	34.0±7.4	32.2 (28.4-36.2)	34.0±6.1	32.9 (30.5-37.3)	0.604
TBW	39±7.5	37.0 (33.5-42.5)	40.4±7.6	40.7 (34.8-44.5)	0.267
FM	36.8±15.2	33.9 (25.0-43.5)	37.5±14.9	35.8 (29.9-41.3)	0.672
FFM	53.1±10.1	50.2 (45.7-57.7)	54.8±10.3	55.3 (47.3-60.2)	0.293
SMM	28.8±6.0	26.9 (24.9-31.5)	29.5±5.8	29.5 (25.4-32.5)	0.327
TM	51.4±1 0.3	52.5 (46.0-55.1)	41.4±8.4	39.0 (36.3-46.5)	**<0.001**
RELATIVE LOSSES					
Dif BMI	12.6±5.3	13.2 (9.40-15.45)	11.0±4.3	9.9 (8.6-13.8)	0.098
Dif TBW	4.1±3.5	3.9 (1.65-6.55)	2.8±4	2.1 (.20-5.3)	0.052
Dif FM	28.2±11.5	29.7 (21.0-34.0)	26.9±10.1	26.6 (19.3-33.5)	0.442
Dif FFM	5.3±4.6	5.3 (2.05-8.65)	3.6±5.2	2.8 (.1-7.3)	0.056
Dif SMM	4.0±2.8	4.0 (2.15-5.6)	3.00±2.9	2.1 (.9-5.4)	0.051
Dif TM	3.0±8.5	0.0 (.00-1.80)	12.7±6.5	12.7 (9.6-17.1)	**<0.001**

*p*-values were calculated using the Mann-Whitney U-test.

BMI, body mass index; FFM, fat-free mass; FM, fat mass; IQR, interquartile range; SMM, skeletal muscle mass; TBW, total body water; TM, trunk mass

## References

[1] Ogurtsova K, da Rocha Fernandes JD, Huang Y, Linnenkamp U, Guariguata L, Cho NH (2017). IDF Diabetes Atlas: Global estimates for the prevalence of diabetes for 2015 and 2040. Diabetes Res Clin Pract.

[2] Schauer PR, Kashyap SR, Wolski K, Brethauer SA, Kirwan JP, Pothier CE (2012). Bariatric surgery versus intensive medical therapy in obese patients with diabetes. N Engl J Med.

[3] Jeon JY, Kim DJ, Ko SH, Kwon HS, Lim S, Choi SH (2014). Current status of glycemic control of patients with diabetes in Korea: the fifth Korea national health and nutrition examination survey. Diabetes Metab J.

[B04] Díaz-Cerezo S, Romera I, Sicras-Mainar A, López-Simarro F, Dilla T, Artime E (2020). Resource use and costs in patients with poorly controlled type 2 diabetes mellitus and obesity in routine clinical practice in Spain. Curr Med Res Opin.

[5] Maegawa H, Ishigaki Y, Langer J, Saotome-Nakamura A, Andersen M; (2021). Japan Diabetes Clinical Data Management (JDDM) Study Group. Clinical inertia in patients with type 2 diabetes treated with oral antidiabetic drugs: Results from a Japanese cohort study (JDDM53). J Diabetes Investig.

[6] Dantas WS, Roschel H, Murai IH, Gil S, Davuluri G, Axelrod CL (2020). Exercise-Induced Increases in Insulin Sensitivity After Bariatric Surgery Are Mediated By Muscle Extracellular Matrix Remodeling. Diabetes.

[7] Rubino F, Nathan DM, Eckel RH, Schauer PR, Alberti KG, Zimmet PZ (2016). Metabolic Surgery in the Treatment Algorithm for Type 2 Diabetes: A Joint Statement by International Diabetes Organizations. Diabetes Care.

[B08] Batterham RL, Cummings DE (2016). Mechanisms of Diabetes Improvement Following Bariatric/Metabolic Surgery. Diabetes Care.

[9] De Luca M, Angrisani L, Himpens J, Busetto L, Scopinaro N, Weiner R (2016). Indications for Surgery for Obesity and Weight-Related Diseases: Position Statements from the International Federation for the Surgery of Obesity and Metabolic Disorders (IFSO). Obes Surg.

[10] Fernandes G, Santo MA, Crespo AFCB, Biancardi GB, Mota FC, Antonangelo L (2019). Early glycemic control and incretin improvement after gastric bypass: the role of oral and gastrostomy route. Surg Obes Relat Dis.

[11] Dirksen C, Jørgensen NB, Bojsen-Møller KN, Jacobsen SH, Hansen DL, Worm D (2012). Mechanisms of improved glycaemic control after Roux-en-Y gastric bypass. Diabetologia.

[12] Still CD, Wood GC, Benotti P, Petrick AT, Gabrielsen J, Strodel WE (2014). Preoperative prediction of type 2 diabetes remission after Roux-en-Y gastric bypass surgery: a retrospective cohort study. Lancet Diabetes Endocrinol.

[13] Aron-Wisnewsky J, Sokolovska N, Liu Y, Comaneshter DS, Vinker S, Pecht T (2017). The advanced-DiaRem score improves prediction of diabetes remission 1 year post-Roux-en-Y gastric bypass. Diabetologia.

[14] Lee WJ, Hur KY, Lakadawala M, Kasama K, Wong SK, Chen SC (2013). Predicting success of metabolic surgery: age, body mass index, C-peptide, and duration score. Surg Obes Relat Dis.

[15] Lee WJ, Almulaifi A (2015). Recent advances in bariatric/metabolic surgery: appraisal of clinical evidence. J Biomed Res.

[16] Aminian A, Brethauer SA, Andalib A, Nowacki AS, Jimenez A, Corcelles R (2017). Individualized Metabolic Surgery Score: Procedure Selection Based on Diabetes Severity. Ann Surg.

[17] Bhasker AG, Remedios C, Batra P, Sood A, Shaikh S, Lakdawala M (2015). Predictors of Remission of T2DM and Metabolic Effects after Laparoscopic Roux-en-y Gastric Bypass in Obese Indian Diabetics-a 5-Year Study. Obes Surg.

[B18] Park JY, Kim YJ (2016). Prediction of Diabetes Remission in Morbidly Obese Patients After Roux-en-Y Gastric Bypass. Obes Surg.

[19] Yan W, Bai R, Yan M, Song M (2017). Preoperative Fasting Plasma C-Peptide Levels as Predictors of Remission of Type 2 Diabetes Mellitus after Bariatric Surgery: A Systematic Review and Meta-Analysis. J Invest Surg.

[20] Tharakan G, Scott R, Szepietowski O, Miras AD, Blakemore AI, Purkayastha S (2017). Limitations of the DiaRem Score in Predicting Remission of Diabetes Following Roux-En-Y Gastric Bypass (RYGB) in an ethnically Diverse Population from a Single Institution in the UK. Obes Surg.

[21] Park JY (2018). Prediction of Type 2 Diabetes Remission after Bariatric or Metabolic Surgery. J Obes Metab Syndr.

[22] Purnell JQ, Selzer F, Wahed AS, Pender J, Pories W, Pomp A (2016). Type 2 Diabetes Remission Rates After Laparoscopic Gastric Bypass and Gastric Banding: Results of the Longitudinal Assessment of Bariatric Surgery Study. Diabetes Care.

[B23] Panunzi S, Carlsson L, De Gaetano A, Peltonen M, Rice T, Sjöström L (2016). Determinants of Diabetes Remission and Glycemic Control After Bariatric Surgery. Diabetes Care.

[24] Still CD, Benotti P, Mirshahi T, Cook A, Wood GC (2019). DiaRem2: Incorporating duration of diabetes to improve prediction of diabetes remission after metabolic surgery. Surg Obes Relat Dis.

[25] Ahuja A, Tantia O, Chaudhuri T, Khanna S, Seetharamaiah S, Majumdar K (2018). Predicting remission of diabetes post metabolic surgery: a comparison of ABCD, diarem, and DRS scores. Obes Surg.

[26] Blackstone R, Bunt JC, Cortés MC, Sugerman HJ (2012). Type 2 diabetes after gastric bypass: remission in five models using HbA1c, fasting blood glucose, and medication status. Surg Obes Relat Dis.

[27] Dixon JB, Chuang LM, Chong K, Chen SC, Lambert GW, Straznicky NE (2013). Predicting the glycemic response to gastric bypass surgery in patients with type 2 diabetes. Diabetes Care.

[28] Aarts EO, Janssen J, Janssen IM, Berends FJ, Telting D, de Boer H (2013). Preoperative fasting plasma C-peptide level may help to predict diabetes outcome after gastric bypass surgery. Obes Surg.

[29] Takesian M, Santo MA, Gadducci AV, Santarém GCF, Greve J, Silva PR (2018). TRUNK BODY MASS INDEX: A NEW REFERENCE FOR THE ASSESSMENT OF BODY MASS DISTRIBUTION. Arq Bras Cir Dig.

[30] Sjöström L (2013). Review of the key results from the Swedish Obese Subjects (SOS) trial - a prospective controlled intervention study of bariatric surgery. J Intern Med.

[31] Belligoli A, Bettini S, Segato G, Busetto L (2020). Predicting Responses to Bariatric and Metabolic Surgery. Curr Obes Rep.

[32] Pajecki D, Halpern A, Cercato C, Mancini M, de Cleva R, Santo MA (2013). Short-term use of liraglutide in the management of patients with weight regain after bariatric surgery. Rev Col Bras Cir.

